# Exploring the Core Attributes of Quality of Life Among Low-Income Terminal Cancer Patients in China: A Network Analysis

**DOI:** 10.3390/healthcare13131521

**Published:** 2025-06-26

**Authors:** Ying Chen, Guojuan Chen, Jianwei Zheng, Yitao Wei, Hong Wu, Huimin Xiao

**Affiliations:** 1 School of Nursing, Xiamen Medical College, Xiamen 361023, China; chenying_yc@126.com (Y.C.);; 2 School of Nursing, Fujian Medical University, Fuzhou 350122, China; 3 Department of Oncology, Fujian Medical University Union Hospital, Fuzhou 350001, China; 4 Department of Hospice Care, Fujian Provincial Hospital, Fuzhou 350001, China; 5 Research Center for Nursing Humanity, Fujian Medical University, Fuzhou 350122, China

**Keywords:** low-income, quality of life, cancer, core attribute, network analysis

## Abstract

Background/Objectives: Increasing evidence has shown that terminal cancer patients experience a poor quality of life (QoL), but the complex internal structure of the QoL among terminal cancer patients is not well documented. This study aimed to explore the core attributes of QoL and their interrelationships in low-income terminal cancer patients stratified by survival time (<3 months vs. 3–6 months). Methods: This study retrospectively analyzed the records of 5649 low-income terminal cancer patients from a hospital hospice center. The Cancer Pain and Quality of Life Questionnaire for Chinese Cancer Patients (CPQLQ) was employed to assess the QoL. A network analysis was conducted to examine centrality indices and density measures. Results: For patients with a survival time of 3 to 6 months, the highest centrality score was associated with “attitudes towards treatment” of the CPQLQ (rs = 1.84). In contrast, for those with a survival time of less than 3 months, “illness perception” of the CPQLQ had the highest centrality score (rs = 1.70). In both the less than 3 months and 3–6 months survival time groups, the network analysis indicated that the strongest correlations were between “illness perception” and “attitudes towards treatment” of the CPQLQ. Conclusions: Attitudes towards treatment and illness perception emerge as core attributes of the QoL network and are strongly interrelated among low-income terminally ill cancer patients. The findings highlight that a shift in hospice care priorities, linked to survival time, is crucial for enhancing the QoL of terminal cancer patients.

## 1. Introduction

Cancer constitutes a major societal problem, a challenge to public health, and a financial strain, responsible for 16.8% of all global fatalities and 22.8% of fatalities from non-communicable diseases [1]. The global cancer statistics for 2022 indicate nearly 20 million new cases and roughly 10 million deaths related to cancer [1]. Of these, 80% of cancers are diagnosed at an advanced or late stage [2]. Advanced or terminal cancer not only significantly affects a patient’s quality of life (QoL), but also adds financial and emotional strain to families [3,4]. Consequently, improving the QoL has become a critical healthcare priority among terminal cancer patients.

QoL is recognized as a fundamental aspect of human existence and is consistently acknowledged as the primary goal of healthcare [5,6]. It covers a range of domains, including physical, mental, spiritual, and societal facets [7]. The World Health Organization defines QoL as “individuals’ perceptions of their position in life in the context of the culture and value systems in which they live and in relation to their goals, expectations, standards, and concerns.” [8,9]. The QoL of cancer patients has always been a concern in the academic community. A meta-analysis of chronic cancer survivors showed that QoL remains considerably impaired for up to 26 years [4]. Furthermore, studies indicate that uncontrolled pain, anxiety, demoralization, spiritual distress, and inadequate end-of-life care contribute to the diminished QoL in this population [10,11,12,13]. Spiritual well-being facilitates acceptance and meaning-making, thereby alleviating despair. Religious and philosophical frameworks also provide narratives that help individuals cope with death anxiety. Collectively, these factors demonstrate the distinct impact of spirituality on the QoL for individuals in terminal stages [14].

Regarding influencing factors of the QoL in cancer patients at the terminal stage, family economic conditions have attracted considerable research attention. Family economic status is identified as a prominent factor affecting the QoL across various types of cancer [15,16]. Kankeu and Parikh-Patel have found that terminal cancer patients from lower-income families tend to have a poorer QoL, compared to those from higher-income families [17,18]. An et al. [19] have conducted a retrospective analysis of the medical records from 2048 cancer patients admitted to hospice care, revealing that physical symptoms significantly burden the patients from low-income families, and improvements in the QoL were urgently needed.

Theoretically, socioeconomic status serves as a fundamental cause of health disparities, shaping access to resources and constraining coping mechanisms. This, in turn, influences the QoL experiences of terminally ill patients [20]. However, the intricate internal structure of QoL, especially among low-income terminal cancer patients, is still poorly understood. Specifically, QoL is a dynamic system that comprises interacting physical, psychosocial, and spiritual components. A systems perspective suggests that QoL emerges from feedback loops between these components, yet these interactions are poorly understood. Therefore, this study aimed to identify the core attributes of QoL and illustrate their interrelationships within this vulnerable population across distinct survival times, from the perspective of network analysis.

## 2. Materials and Methods

### 2.1. Design

This was a retrospective study that employed network analysis. Network analysis is an approach that enables quantitative explanation and visualization of the interrelationships among various variables [21]. It posits that psychological constructs arise from complex systems generated by the interactions among their components, offering a fresh perspective for identifying core psychological symptoms [22,23,24]. Previous studies have demonstrated that it is logical and practical to characterize a variable as a complex system resulting from the interactions between its components [25]. Network analysis is rooted in complex systems theory [24]. It conceptualizes QoL not as a latent trait but as an emergent system of interacting elements, where symptoms, perceptions, and functional abilities mutually influence each other. In this study, network analysis was used to explore the network structure of QoL and identify its core attributes, which refer to nodes with the highest centrality within the network structure.

### 2.2. Data Collection

The data were collected retrospectively from the existing medical records at the Hospice Center of Fujian Provincial Hospital in Fuzhou, China. This hospice center, a non-profit institution, only offers home-based palliative care for low-income patients with terminal cancer in Fujian. Low-income refers to households meeting either per capita income below 1.5 times the locally specified minimum living allowance standard, or per capita income below the previous year’s local per capita disposable income, with essential expenditures exceeding a locally defined proportion of total household income. Those records were included if the patients were ① diagnosed with terminally ill cancer, ② had a survival time of six months or less (calculated from the patient’s initial admission to the time of death), and ③ were aged 18 years or older.

Patient records were collected from January 2001 through December 2020. These records covered a comprehensive assessment of patients at the first home visits, as well as all subsequent follow-up visits until services were discontinued or the patient passed away.

### 2.3. Measures

#### 2.3.1. Sociodemographic and Clinical Characteristics

During the initial home visits, hospice nurses documented the patients’ sociodemographic and clinical profiles. Sociodemographic information included gender, age, ethnicity, educational level, and histories of alcohol and tobacco use. Clinical data related to the disease included the type of cancer, history of surgery, chemotherapy, or radiotherapy, comorbidities, survival duration, and Karnofsky Performance Status (KPS).

#### 2.3.2. Quality of Life

The Cancer Pain and Quality of Life Questionnaire for Chinese Cancer Patients (CPQLQ; Supplementary Table S1) was routinely used to assess patients’ QoL by the study hospice center during 2001–2020. This 12-item questionnaire, rated on a 5-point Likert scale, assesses aspects such as appetite, mental state, sleep, fatigue, pain, family and work relationships, illness perception, attitudes towards treatment, daily activities, treatment side effects, and facial expressions. The total score ranges from 12 to 60, where higher scores suggest a better QoL. The CPQLQ demonstrated good reliability (Cronbach’s α = 0.862) [26]. It has been widely adopted in Chinese hospice centers since its development, ensuring contextual appropriateness [26]. Self-report QoL data were collected by well-trained hospice nurses at the first home visit.

### 2.4. Data Analysis

Descriptive analyses and an undirected network analysis were conducted using R 4.4.1 software. To ensure the reliability of the network analysis, cases with missing scores for two or more items in the CPQLQ were excluded, leaving 5649 cases for the final analysis. The MissForest algorithm was utilized to impute the missing data (Supplementary Table S2 and Figure S1) [27]. Descriptive statistics were employed to profile the demographic and clinical variables, as well as the scores of the CPQLQ. The stratification of survival time (<3 months vs. 3–6 months) was based on robust clinical evidence of functional and symptomatic trajectories at end-of-life. Specifically, Lunney et al.’s [28] analysis of 144,456 cancer decedents revealed that functional status remains relatively preserved until 5–6 months before death but undergoes a precipitous decline within the final 3 months. This aligns with Li et al.’s [29] findings that symptom burden escalates severely in the last 3-month period, critically disrupting daily functioning. These patterns indicate a biological inflection point at 3 months. This is supported by international guidelines, which define the 3–6 month period as the palliative intervention window and the <3 month period as the terminal phase requiring hospice-focused care. Therefore, two survival time categories (<3 months vs. 3–6 months) were employed to stratify the analysis.

#### 2.4.1. Network Estimation

The Qgraph module was utilized to construct and visualize the network using the Fruchterman-Reingold algorithm [30,31]. In the network, nodes represent the diverse items of the CPQLQ. Edges, on the other hand, depict the partial correlation coefficients between these items, with thicker lines signifying stronger interconnections. The least absolute shrinkage and selection operator (LASSO) was employed as a regularization technique to minimize small edges to zero, thereby reducing false-positive connections from the model as a regularization technique [32]. The extended Bayesian information criterion (EBIC) was optimized with a tuning parameter at 0.5 [33].

Furthermore, we conducted a multilinear regression analysis to evaluate the statistical relevance of sociodemographic and clinical factors associated with CPQLQ scores. Several factors were identified as significant (*p* < 0.05), including gender, education level, cancer type, history of surgery, chemotherapy, radiotherapy, comorbidities, alcohol and tobacco use, survival time, and KPS. These factors were selected a priori as covariates to investigate the interrelationships among the 12 items of the CPQLQ. All categorical variables were appropriately transformed into numerical representations for regression analysis, using dummy coding for nominal variables and ordinal coding for ordered categories. The complete coding scheme is presented in Supplementary Table S3.

#### 2.4.2. Centrality Measurements

In our study, we calculated the strength centrality metric to identify the most central node [34]. Strength represents the sum of absolute edge weights connected to a node, reflecting its potential to activate other nodes when activated [35]. Strength centrality is more reliable in psychological networks because it directly quantifies the potential influence or activation capacity of a node within the network. Higher strength centrality indicates stronger connections to other nodes, implying greater capacity to influence and be influenced by them. Prior studies have demonstrated that two alternative centrality metrics, namely betweenness and closeness, lack the stability required for assessing node centrality [36], as their calculations are sensitive to minor network perturbations, potentially compromising result reproducibility. Therefore, we selected strength centrality for its robust and interpretable measurement of nodal influence.

#### 2.4.3. The Estimation of Network Accuracy and Stability

We evaluated the accuracy of network connections using 95% bootstrap confidence intervals (CIs) for the weights of those connections. The accuracy of edge estimates increases with decreasing CI overlap. Subsequently, we employed the case-dropping subset bootstrap to determine the centrality stability (CS) coefficient, evaluating the robustness of strength centrality. The CS coefficient indicates the maximum proportion of cases that can be omitted while maintaining a 95% probability that the correlation between the initial sample’s strength and bootstrapped subsets is at least 0.7 [37]. Ideally, the CS coefficient should exceed 0.25, with 0.50 being preferred.

#### 2.4.4. Network Comparison Test

To assess the differences between group networks, network comparison tests (NCT) were performed using the R package (1.2.1) Network Comparison Test. This two-tailed permutation test compares discrepancies between two networks in terms of network topology, overall strength, and edge strength [38]. To address the influence of sample size imbalance between groups on the power of the NCT, a random sample was drawn from the larger group (0–3 months) to align with the size of the smaller group (3–6 months). The NCT results were presented as mean invariance statistics and *p*-values.

#### 2.4.5. Sensitivity Analysis

Sensitivity analyses were conducted to assess the stability of the network findings. First, we employed the MissForest algorithm to impute missing data. Secondly, given the extensive data collection period (nearly 20 years), these factors may affect the stability of network analysis results. Therefore, we implemented two sensitivity analysis approaches to test the stability of network analysis: (1) conducting network analysis on the complete dataset prior to imputation; (2) performing network analysis on the datasets before and after 2017, which coincided with the initiation of a nationwide pilot project on hospice care by the National Health Commission of the People’s Republic of China.

### 2.5. Ethics Consideration

This study was authorized by the Fujian Provincial Hospice Center and received approval from the Ethics Committee of Fujian Medical University (Authorization no. 2022–91). Due to the study’s retrospective nature, the Ethics Committee waived the requirement for written informed consent from participants. However, we adhered to strict ethical principles throughout the research process. To protect patient privacy and confidentiality, all patient records were anonymized before analysis, with personal identifiers removed and replaced with unique codes. Only aggregated data were used for statistical analysis, ensuring that no individual patient information could be traced back to a specific person. We also ensured that the data collected and used in this study were handled in accordance with the relevant guidelines and regulations, including the Declaration of Helsinki. The research team members were trained in research ethics and followed ethical guidelines rigorously during all stages of the study.

## 3. Results

### 3.1. Participants

The study ultimately included 5649 hospice care patients. Their sociodemographic and clinical characteristics are outlined in Table 1. Most participants were male (n = 3385, 59.9%), of Han ethnicity (n = 4323, 76.5%), married (3453, 61.1%), and had a primary school education level or below (n = 3649, 64.6%). Gastrointestinal cancer was the most prevalent cancer among the patients (2617, 46.3%), followed by lung cancer (n = 1683, 29.8%). The median survival time was 40.0 days. Regarding the history of therapy, most patients received no therapy (2242, 39.7%). Most patients had comorbidities (4269, 75.6%), a history of alcohol and tobacco (4531, 80.2%), and poor physical status (n = 4640, 82.1%).

### 3.2. Estimated QoL Networks

**Figure 1:**
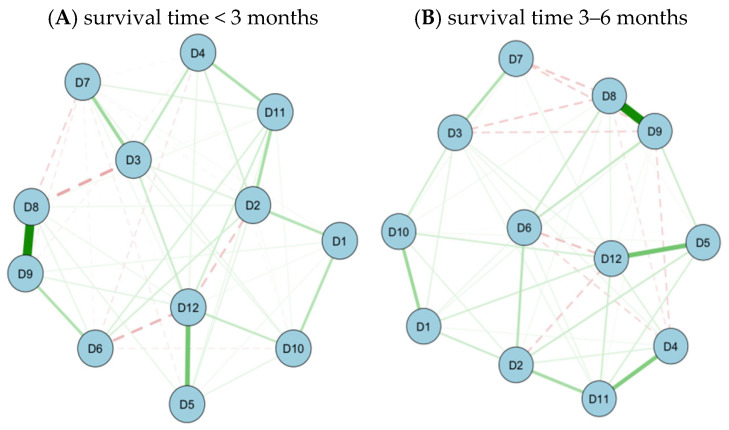
Estimated QoL networks. Note: Solid lines depict positive correlations, dashed lines represent negative correlations, and thick lines denote strong connections among nodes. Green edges represent positive relationships, while red edges denote negative ones. D1: Appetite; D2: Mental; D3: Sleep; D4: Fatigue; D5: Pain; D6: Family relationships; D7: Work relationships; D8: Illness perception; D9: Attitudes toward treatment; D10: Activities of daily life; D11: Treatment related side effect; D12: Facial expression.

The diagram in Figure 1 illustrates the network configurations of QoL for patients categorized by their survival durations. Among those with a survival period shorter than 3 months, the pair exhibiting the most robust positive correlations was determined to be illness perception and attitudes towards treatment. Similarly, for patients who survived between 3 and 6 months, this pair also demonstrated the strongest positive connections.

### 3.3. Centrality Indices

**Figure 2:**
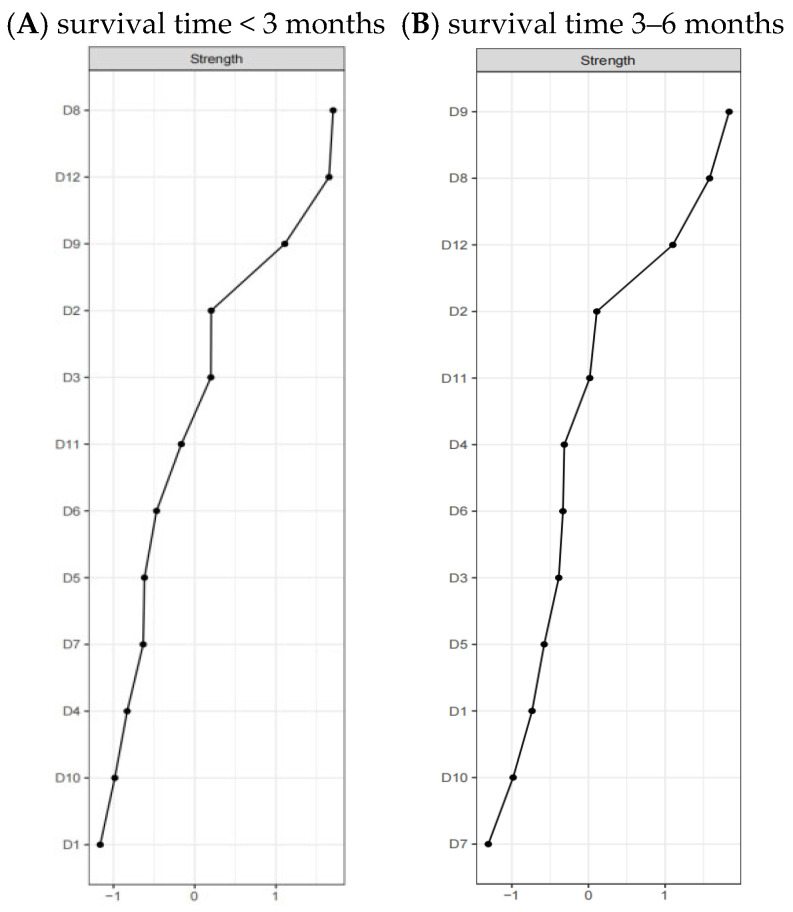
Comparison of network centrality indices.

The normalized measures of centrality indices for the QoL networks that correspond to varying survival durations are displayed in Figure 2. Specifically, in the network for patients with a survival time of less than 3 months, illness perception exhibited the highest level of strength centrality, with a correlation coefficient (rs) of 1.70. In contrast, for the network representing patients with a survival period of 3 to 6 months, the attitudes towards treatment achieved the highest strength centrality, marked by an rs value of 1.84.

### 3.4. Network Accuracy and Stability

**Figure 3:**
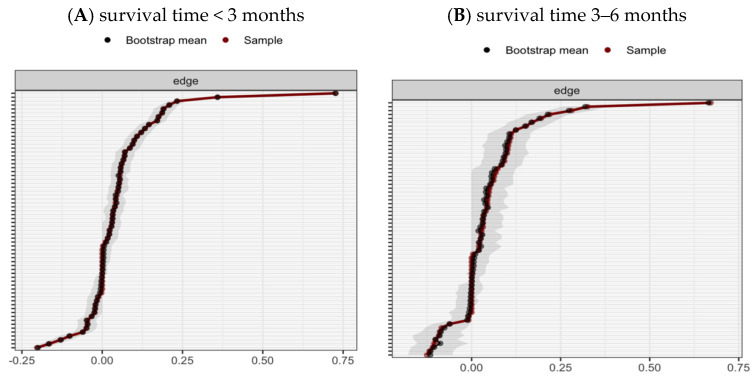
Bootstrapped 95% confidence intervals of edge weights.

**Figure 4:**
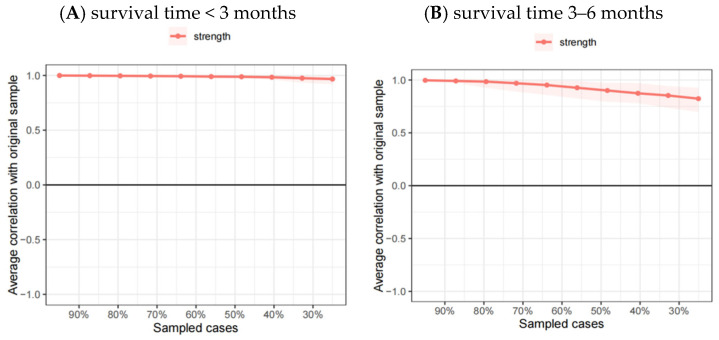
Stability of the centrality indices.

The 95% confidence intervals (CIs) for edge weights, obtained through bootstrapping, are depicted in Figure 3. The bootstrap CIs for both groups indicate that these two networks are reliable. The CS coefficient was 0.75 for the network comprising patients with a survival time of less than 3 months, and it was 0.67 for the network of patients who survived between 3 and 6 months (see Figure 4). For estimation of edge weight differences and node strength differences, see Supplementary Figures S2 and S3.

### 3.5. Survival Time-Network Comparison

To statistically compare the QoL network structures between survival groups, we conducted an NCT. The results indicated no significant differences in global network strength (test statistic S = 0.316, *p* = 0.453) or network structure invariance (test statistic M = 0.116, *p* = 0.368) between patients with survival times of less than 3 months and those between 3 and 6 months.

### 3.6. Results of Sensitivity Analysis

Two sensitivity analysis approaches were used, and both identified the same core attributes. Detailed results of the sensitivity analyses are presented in Supplementary Figures S4–S12.

## 4. Discussion

This study employed network analysis to identify the core attributes of QoL and their interrelationships among low-income terminal cancer patients in China. Our findings reveal a significant shift in the core attribute of QoL: attitudes towards treatment exhibited the highest centrality for patients with 3–6 months survival, while illness perception was the most central attribute for those with <3 months of survival. Crucially, the strongest pairwise association in both groups existed between illness perception and attitudes towards treatment. These findings demonstrate distinct network structures of QoL associated with different survival periods, and the relative interconnectedness of specific QoL attributes varies based on survival time within this vulnerable population.

The study findings indicate that attitudes towards treatment emerged as a core attribute in the QoL network of low-income patients with terminal cancer, whose survival period is 3 to 6 months. Attitudes towards treatment refer to the patient’s perception, emotional response, and intentional behavior toward the treatment process and its potential outcomes [39]. Low-income patients with terminal cancers frequently experience complex physiological and psychological challenges, including pain, malnutrition, anemia, anxiety, and depression [19,40]. A positive attitude towards treatment, such as having confidence and actively cooperating, fosters greater adherence to care plans and active engagement in symptom management. This not only improves procedural compliance but also cultivates a sense of agency and hope. For instance, patients with positive attitudes are more likely to follow medical advice, enhancing symptom control and psychological comfort. Conversely, negative attitudes (e.g., hopelessness or skepticism toward treatment) correlate with reduced adherence and poorer QoL. Thus, attitudes toward treatment function as a structurally central hub, likely interacting bidirectionally with other QoL domains. While centrality denotes topological importance, causal directionality cannot be inferred from our design. In clinical practice, prioritizing assessment and cultivation of positive attitudes during this phase is essential. Strategies include targeted psychological support, clear communication about realistic treatment goals, and collaborative decision-making to bolster perceived control.

In contrast, this study revealed a significant shift when the survival time is reduced to less than three months: illness perception emerges as the core attribute of QoL. Illness perception, a critical concept in psycho-oncology, refers to patients’ views, beliefs, and emotional reactions to their illness and treatment [41]. As the disease progresses to a more critical stage, the patient’s understanding and acceptance of their condition, along with their emotional responses, become the dominant factors influencing their survival experience. Previous studies have demonstrated that illness perception is linked to the QoL of cancer patients. For instance, Cheng et al. [42] found that illness perception significantly influences fear of progression in patients with digestive system cancers, highlighting its role in shaping patients’ psychological responses to their condition. Similarly, Zheng et al. [43] reported that illness perception, in conjunction with cognitive emotion regulation strategies, affects psychological distress in breast cancer patients and their spouses. These findings align with our results, underscoring the importance of illness perception in the QoL of cancer patients. According to Omiaowska et al., cancer patients with a positive view of their illness report better QoL and experience less severe symptoms related to their cancer and treatment [44]. Compared to patients with a survival time of 3 to 6 months, those with less than 3 months of survival face a continuous worsening of physical symptoms. As the disease becomes more alarming and threatening, patients often develop a more negative illness perception. At this stage, they may confront more life-and-death decisions and experience heightened fear and anxiety, making a correct understanding of their disease and psychological adjustment particularly critical. Çam et al. [45] also emphasized that negative illness perceptions can predict hopelessness and death anxiety in palliative care patients, further supporting the significance of addressing illness perception in end-of-life care. Low-income groups, in particular, tend to have limited access to healthcare and financial resources, which reduces their access to disease-related information [46,47]. This information asymmetry further exacerbates their fear and anxiety about their condition.

The results of this research indicate a strong association between illness perception and attitudes towards treatment within the QoL network of low-income terminal cancer patients, observed consistently across survival periods of both less than 3 months and 3–6 months. This finding aligns with previous research demonstrating a positive link between illness perception and treatment adherence [48,49]. Illness perception—encompassing a patient’s subjective understanding of disease severity, treatment expectations, and psychological burden—likely shapes their attitudes towards treatment choices. Consequently, this significant association persists regardless of survival time in this population. Furthermore, the limited economic resources of low-income patients present additional challenges in accessing comprehensive healthcare and support, potentially intensifying the complexity of this relationship between perception and attitude.

### Strengths and Limitations

This study has several notable strengths. First, it included 5649 patients with terminal-stage cancer, a relatively large sample size that enhances the reliability of the research findings. This extensive data foundation allows for a more accurate analysis of the complex relationships between various aspects of QoL among low-income patients with terminal cancer. Additionally, sensitivity analyses confirmed the robustness of the results, demonstrating the stability of the network structure.

However, there are certain limitations in this study. First, the data spanned a lengthy time frame, which may introduce potential biases as policies and clinical practices may change over time. Significant policy milestones, such as the 2017 nationwide hospice care pilot, likely improved access to palliative resources for low-income patients in later years. Likewise, clinical advancements and socioeconomic changes may have influenced symptom burden and financial toxicity. Our sensitivity analysis comparing pre- and post-2017 cohorts demonstrated stability in core network attributes, supporting the validity of our primary conclusions. However, temporal variations in peripheral connections may still exist and could potentially affect the results. Meanwhile, the retrospective nature of data collection may lead to incomplete information or inaccurate records, which could impact the overall accuracy of the results. Future longitudinal studies should explicitly model era effects to better disentangle cohort-specific dynamics. Second, our sample was drawn from a specific area, which may restrict the generalizability of our findings to other regions or countries due to different healthcare systems and cultural factors. Future studies with broader geographic representation could enhance the universality of the findings in this field. Third, the scale used to assess QoL in this study has limited international application, which restricts the generalizability of our findings to other cultural contexts and hinders direct comparisons with studies conducted in different regions. Fourth, while network centrality identifies nodes with high structural connectivity, it does not imply causal relationships. Our cross-sectional data cannot establish directional relationships among QoL attributes. Future studies with longitudinal or experimental designs are needed to test causal pathways.

## 5. Conclusions

Using network analysis, this study identified distinct patterns in the interconnected structure of QoL among low-income terminal cancer patients at different survival periods. For patients with a survival time of 3–6 months, attitudes towards treatment emerged as the node with the highest centrality within the QoL network. Conversely, for patients with a survival expectancy of less than 3 months, illness perception demonstrated the highest centrality. Crucially, the strongest pairwise association in both groups linked illness perception and attitudes towards treatment. Despite its retrospective design, the findings suggest that health providers could target illness perceptions and treatment attitudes in order to enhance the QoL of this vulnerable group.

## Figures and Tables

**Table 1:** Characteristics of patients (n = 5649).

Characteristics	n (%), M (P25, P75)
Age	63.0 (54.0, 72.0)
Gender	
Male	3385 (59.9)
Female	2264 (40.1)
Ethnicity	
Han	4323 (76.5)
Minority	1325 (23.5)
Marital status	
Married	3453 (61.1)
Single	2196 (38.9)
Education level	
Primary school or below	3649 (64.6)
Middle school	1363 (24.1)
High school or above	637 (11.3)
Type of cancer	
Gastrointestinal cancer	2617 (46.3)
Lung cancer	1683 (29.8)
Liver cancer	1094 (19.4)
Breast cancer	332 (5.9)
Others	77 (1.4)
Survival time(day)	40.0 (17.0–76.0)
Kinds of received therapy (Surgery/chemotherapy Radiotherapy)	
0	2242 (39.7)
1	1572 (27.8)
2	1441 (25.5)
3	394 (7.0)
Comorbidities	
Yes	4269 (75.6)
No	1380 (24.4)
History of alcohol and tobacco	
Yes	4531 (80.2)
No	1118 (19.8)
KPS	
0~40	4640 (82.1)
50~100	1009 (17.9)

## Data Availability

The datasets used/analyzed in this study are available from the corresponding author upon request.

## Supplementary Materials

The following supporting information can be downloaded at: https://www.mdpi.com/article/10.3390/healthcare13131521/s1, Table S1: The Cancer Pain and Quality of Life Questionnaire for Chinese Cancer patients. Table S2: Percentage of missing data. Table S3: Independent variable coding. Figure S1: Proportion of missing. Figure S2: Estimation of edge weight difference. Figure S3: Estimation of node strength difference. Figure S4: Estimated QOL networks (network analysis on the complete dataset prior to imputation). Figure S5: Comparison of network centrality indices (network analysis on the complete dataset prior to imputation). Figure S6: Bootstrapped 95% confidence intervals of edge weights (network analysis on the complete dataset prior to imputation). Figure S7: Estimated QOL networks (network analysis on the datasets before 2017). Figure S8: Comparison of network centrality indices (network analysis on the datasets before 2017). Figure S9: Bootstrapped 95% confidence intervals of edge weights (network analysis on the datasets before 2017). Figure S10: Estimated QOL networks (network analysis on the datasets after 2017). Figure S11: Comparison of network centrality indices (network analysis on the datasets after 2017). Figure S12: Bootstrapped 95% confidence intervals of edge weights (network analysis on the datasets after 2017).

## Abbreviations

CIs	Confidence intervals
CPQLQ	Caner Pain and QoL Questionnaire for Chinese Cancer Patients
CS	Centrality stability
EBIC	Extended Bayesian information criterion
KPS	Karnofsky Performance Status
NCT	Network comparison tests
LASSO	Least absolute shrinkage and selection procedure
QoL	Quality of Life
