# Supplementary material for: Exploring the Core Attributes of Quality of Life Among Low-Income Terminal Cancer Patients in China: A Network Analysis
Source: Healthcare (Basel). 2025 Jun 26;13(13):1521. doi: 10.3390/healthcare13131521 (PMC12249182; doi:10.3390/healthcare13131521)
Supplement: Supplementary file 1 [file healthcare-13-01521-s001.zip › Supplementary Figure S7 S8 S9.pdf]

Network analysis on the datasets before 2017

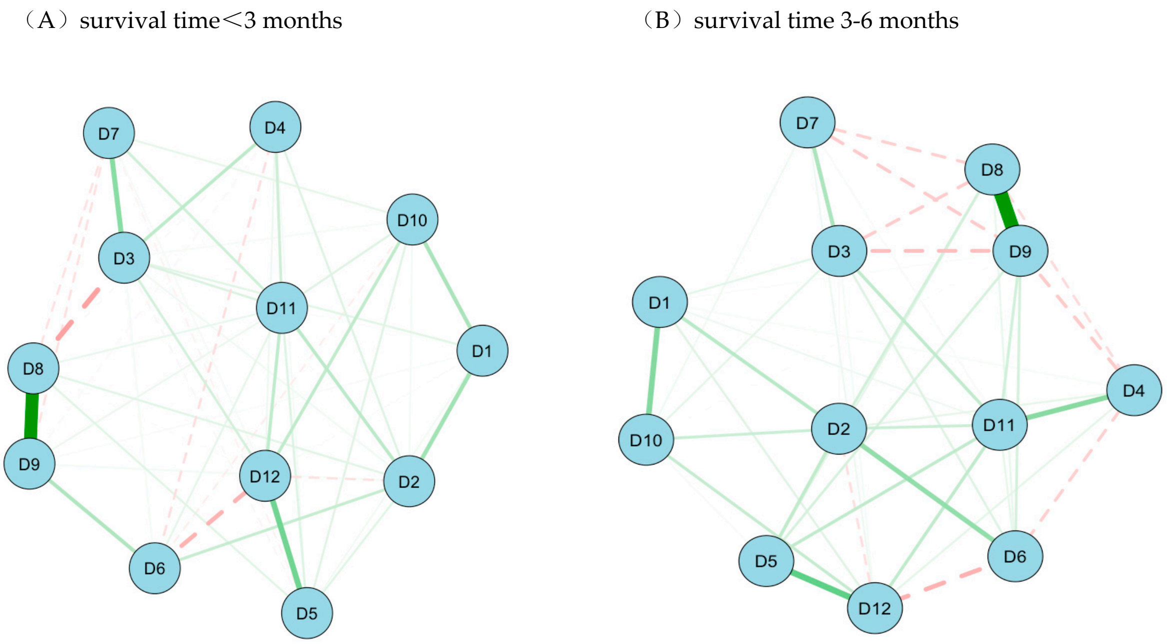

Figure S7. Estimated QOL networks.

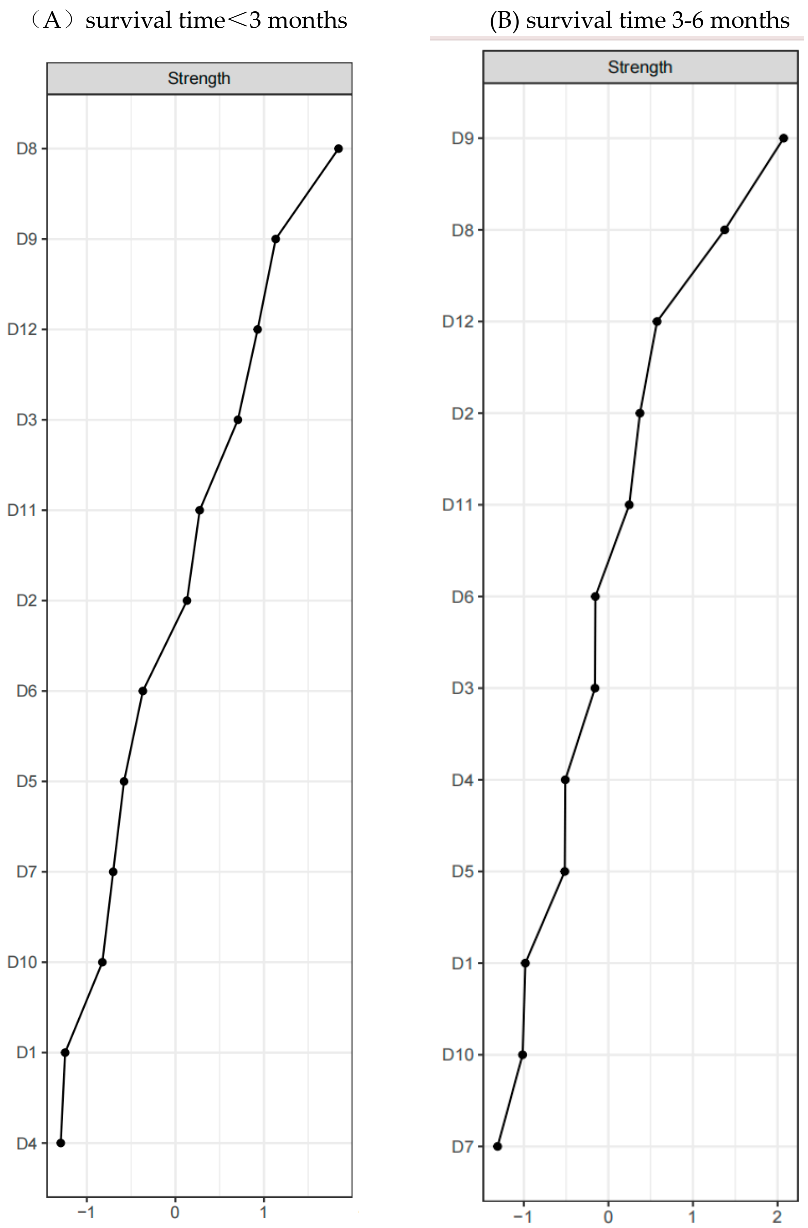

Figure S8. Comparison of network centrality indices

(A) survival time<3 months (B) survival time 3-6 months

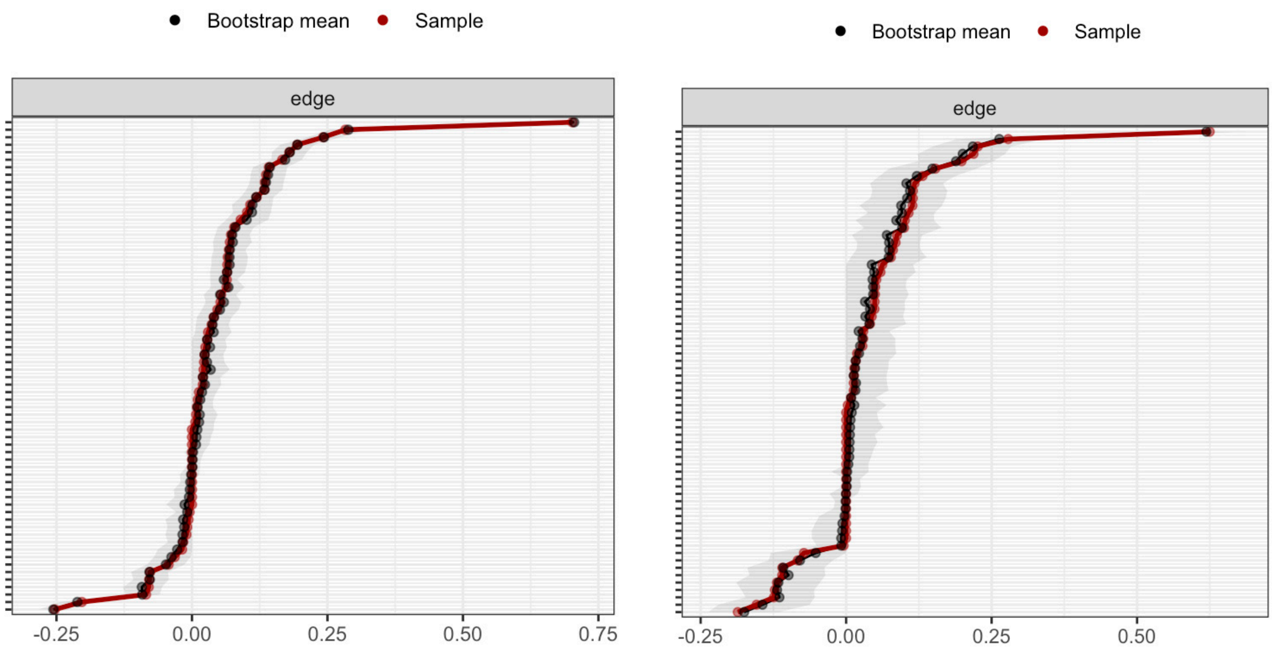

Figure S9. Bootstrapped 95% confidence intervals of edge weights
