# Supplementary material for: Exploring the Core Attributes of Quality of Life Among Low-Income Terminal Cancer Patients in China: A Network Analysis
Source: Healthcare (Basel). 2025 Jun 26;13(13):1521. doi: 10.3390/healthcare13131521 (PMC12249182; doi:10.3390/healthcare13131521)
Supplement: Supplementary file 1 [file healthcare-13-01521-s001.zip › Supplementary Table S1.pdf]

Table S1 The Cancer Pain and Quality of Life Questionnaire for Chinese Cancer patients

Appetite	☐Barely able to eat (1); ☐Food intake < 1/2 normal (2);<br>☐Halve food intake (3); ☑Slightly less food intake (4); ☐Normal (5)
Mental	☐Very poor (1); ☐Poor (2); ☐Sometimes good and sometimes bad (3);<br>☐Ordinary (4); ☐Normal (5)
Sleep	☐Very poor (1); ☐Poor (2); ☐Sometimes good and sometimes bad (3);<br>☐Ordinary (4); ☐Normal (5)
Fatigue	☐Frequent fatigue (1); ☐Feeling powerless (2); ☐Sometimes fatigue (3);<br>☐Sometimes mild fatigue (4); ☐Normal (5)
Pain	☐Severe pain with passive positioning or pain lasting more than 6 months (1);<br>☐Severe pain (2); ☐Moderate pain (3); ☐Mild pain (4); ☐Normal (5)
Family relationships	☐Don't understand at all (1); ☐Poor (2); ☐Ordinary (3);<br>☐Better (4); ☐Good (5)
Work relationships	☐Don't understand and no one takes care of (1); ☐Poor (2); ☐Ordinary (3);<br>☐Few people understand caring (4); ☐Most people understand caring (5)
Illness perception	☐Disappointed, totally uncooperative (1); ☐Uneasy, reluctant to cooperate (2);<br>☐Uneasy, general cooperation (3); ☐Uneasy, but able to cooperate well (4);<br>☐Optimistic, confident (5)
Attitudes towards treatment	☐No hope for treatment (1); ☐Skepticism about treatment (2);<br>☐Hoping to see the curative effect, but afraid of side effects (3);<br>☐Hope to see the curative effect, with general cooperation (4);<br>☐Have confidence, actively cooperate (5)
Activities of daily life	☐Bedrid (1); ☐Can be active, most of the time need to stay in bed (2);<br>☐Active, sometimes bedridden (3); ☐Live a normal life but cannot work (4);<br>☐Normal life and work (5)
Treatment related side effect	☐Seriously affecting daily life (1); ☐Affect daily life (2);<br>☐After symptomatic treatment, daily life can not be affected (3);<br>☐No symptomatic treatment may affect daily life (4); ☐Normal (5)
Facial expression	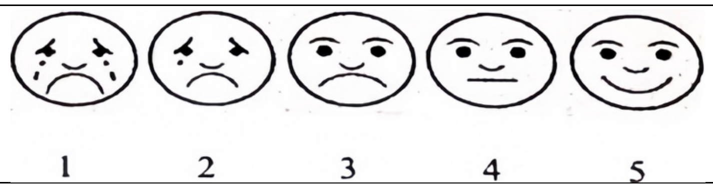
