# Supplementary material for: Exploring the Core Attributes of Quality of Life Among Low-Income Terminal Cancer Patients in China: A Network Analysis
Source: Healthcare (Basel). 2025 Jun 26;13(13):1521. doi: 10.3390/healthcare13131521 (PMC12249182; doi:10.3390/healthcare13131521)
Supplement: Supplementary file 1 [file healthcare-13-01521-s001.zip › Supplementary Table S2.pdf]

Table S2 Percentage of missing data

Varible	Missing percentage(%)
Gender	0
Age	4.23
Ethnicity	0
Surgery	0
Chemotherapy	0
Radiotherapy	0
History of alcohol and tobacco	0
KPS	0
Marital status	2.6
Type of cancer	0.96
Comorbidities	0
Education level	5.91
Survival time	0
D1	0.09
D2	0.09
D3	0.09
D4	0.16
D5	0.07
D6	0.28
D7	18.13
D8	0.32
D9	0.25
D10	0.12
D11	9.29
D12	0.25
