# Supplementary material for: Exploring the Core Attributes of Quality of Life Among Low-Income Terminal Cancer Patients in China: A Network Analysis
Source: Healthcare (Basel). 2025 Jun 26;13(13):1521. doi: 10.3390/healthcare13131521 (PMC12249182; doi:10.3390/healthcare13131521)
Supplement: Supplementary file 1 [file healthcare-13-01521-s001.zip › Supplementary Table S3.pdf]

Table S3 Independent variable coding

Independent Variable	Coding Methodology
Gender	Male=1, Female=0
Marital Status	With spouse=1, Without spouse=0
Ethnicity	Han=1, Other Ethnicities=0
Cancer Type	Reference Category: Gastrointestinal Cancer (x1=0, x2=0)
	• Respiratory Cancer: x1 = 1, x2 = 0
	• Other Cancers: x1 = 0, x2 = 1
Chemotherapy History	Received = 1, Not Received = 0
Radiotherapy History	Received = 1, Not Received = 0
Surgery History	Received = 1, Not Received = 0
Comorbidities	Received = 1, Not Received = 0
Smoking/Alcohol History	Received = 1, Not Received = 0
Education Level	Illiterate = 1, Primary School = 2, Junior High School = 3, High School or Higher = 4
