# Supplementary figures and images for: Exploring the Core Attributes of Quality of Life Among Low-Income Terminal Cancer Patients in China: A Network Analysis
Source: Healthcare (Basel). 2025 Jun 26;13(13):1521. doi: 10.3390/healthcare13131521 (PMC12249182; doi:10.3390/healthcare13131521)

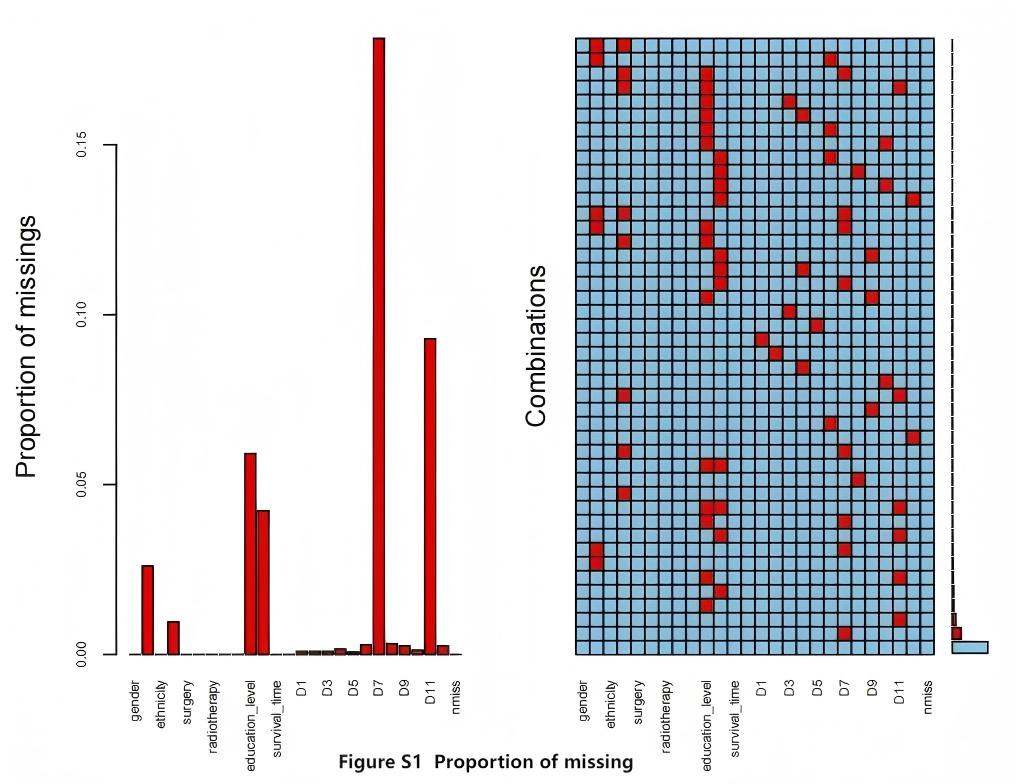

Supplement: Supplementary file 1 [file healthcare-13-01521-s001.zip › Supplementary Figure S1.jpg]
